# The Lymphatic Glands of the Face

**Published:** 1907-11-30

**Authors:** 


### Anatomical Points.

### THE LYMPHATIC GLANDS OF THE FACE.

The lymphatic glands of the face sometimes receive too little attention in text-books of Anatomy, partly, no doubt, on account of their small size. They are of considerable importance in surgery, however, in connection with epitheliomata of the lip or cheek, for example, or with suppurative or other inflammatory lesions of the ear, nose, eye, or face, such as impetigo, eczema that has become pus-inoculated, and so forth. M. Princeteau has made an exhaustive study of these lymphatic glands of the face, both in the cadaver, and in clinical surgery. He describes four distinct groups of them, as follows:—

1. *The buccal group of lymphatic glands.*—These are situated upon the external aspect of the buccinator muscle in front of the facial vein. They vary from one to three in number, and when three are present they are arranged diagonally between the termination of Stenon's duct and the insertion of the buccinator muscle on to the inferior maxilla. They may be mistaken for the buccal salivary glands which are situated very near them.

2. *The supra-maxillary group.*—These also vary from one to three in number. They are situated on the outer aspect of the inferior maxilla; usually they are in direct relationship with the facial vein, but sometimes there are also some around the facial artery; possibly one should distinguish an anterior group related to the artery and a posterior group related to the vein.

3. *The infra-maxillary lymphatic gland.*—This is usually single; it requires a separate place in classification, not only upon anatomical grounds, but also on account of its pathological reactions. It is separate from the submaxillary lymphatic glands, and indeed it is not uncommon to find it upon the inferior margin of the mandible; it may sometimes be moved about by the finger so as to lie alternately upon and beneath the inferior maxilla.

4. *The commissural group.*—This lies upon the outer aspect of the orbicularis oris muscle, about a quarter of an inch behind the angle of the mouth.

None of these lymphatic glands can be palpated in a perfectly healthy person, but it is not at all uncommon to find them enlarged and palpable in inflammatory or malignant affections. The lymphatic gland, which lies just in front of the external auditory meatus over the posterior border of the parotid salivary gland and which is sometimes called the parotid lymphatic gland, is not included in Princeteau's list, because it is so well known, and this observer prefers to draw particular attention to the less known lymphatic glands of the face. It has long been recognised that it is important to note whether the parotid lymphatic gland is involved when there is malignant disease of the skin of the face or ear, for example; and Princeteau points out how involvement of the less known lymphatic glands of the cheek and jaw is of equal clinical importance.

When the glands are inflamed or enlarged, they may attract attention on inspection, but this is the exception rather than the rule; more often they need careful examination for their detection. The buccal group should be palpated bi-digitally, with the index-finger of one hand inside the mouth against the mucous surface of the cheek, and the thumb of the same hand upon the skin outside. The whole of the cheek may thus be palpated step by step. It is particularly near the anterior border of the masseter that the examination should be most thorough. The commissural gland may be palpated in a similar way. The supra- and infra-maxillary lymphatic glands are best examined for by careful palpation of the corresponding regions from outside.

Any one of them, when inflamed, may soften and form an abcess, which always points externally. Apart from malignant disease, the conditions in which enlargement of the glands, with or without suppuration in them, has been noted are: impetigo, impetiginous eczema of the lips and their commissures, of the nares, or of the cheek, lupus of the nose or cheek, syphilitic ulcerations around the mouth, the eruption of teeth, caries of the premolars, otitis media, adenoids, and German measles. Sometimes the glands have been painfully swollen without any discernible cause.

